# Associations Between Parenting Stress and Quality Time in Families of Youth with Autism Spectrum Disorder

**DOI:** 10.1007/s10803-022-05852-0

**Published:** 2023-01-10

**Authors:** Rebecca S. Bradley, Grace L. Staples, Lauren B. Quetsch, Lindsey S. Aloia, Cynthia E. Brown, Stephen M. Kanne

**Affiliations:** 1 University of Arkansas, 316B Memorial Hall, 72701 Fayetteville, AR USA; 2 Pacific University, College Way, 97116 Forest Grove, OR USA; 3 Center for Autism and the Developing Brain, Weill Cornell Medical College, New York, USA

**Keywords:** Autism Spectrum Disorder, Stress, Quality Time, Parent-Child Relationship

## Abstract

Increased stress among parents of youth with ASD has been well-documented. However, research on aspects of the parent-child relationship and subsequent links to parenting stress is limited. We assessed parents (*N* = 511) of youth with ASD to examine relations between parenting stress and parent-child quality time (amount of quality time, shared enjoyment, synchronicity). Elevated parenting stress was associated with less time spent engaging with youth in shared activities and decreased parent and child enjoyment during shared interactions. Parents with elevated stress reported engaging in shared activities and experiencing synchronicity with their child less often than parents below the clinical threshold. Future research should emphasize longitudinal efforts examining the directionality of this relationship to better inform family-focused intervention.

Autism spectrum disorder (ASD) is a neurodevelopmental condition characterized by difficulties in social communication and social interaction, and restricted, repetitive behaviors and interests (American Psychiatric Association, 2013). There is an extensive literature highlighting the significant levels of parenting stress many caregivers of youth with ASD experience (for reviews, see Enea & Rusu, 2020; Hayes & Watson, 2013) and its connection to negative family outcomes including caregiver depressive symptoms, child disruptive behavior, and caregiver overall involvement with their child (Olson et al., 2021; Schiltz et al., 2018; Weitlauf et al., 2014). Recent research has found that negative caregiver psychological wellbeing, including elevated parenting stress, is also associated with lower quality of parent-child relationships (Hickey et al., 2020). Conversely, closeness in the parent-child relationship is associated with positive family outcomes including lower rates of emotional and behavioral dysregulation in children with ASD (Greenlee et al., 2021). Thus, exploring specific factors that may promote positive parent-child relationships and protect against parenting stress is important to better understand how families of youth with ASD spend their time together.

Limited literature exploring caregiver involvement (frequency of parent-child interactions, caregiver awareness of child events/activities) in families of youth with ASD is available (e.g., Schiltz et al., 2018); furthermore, almost no research to date has investigated the presence of enjoyment during caregivers’ time spent with their child with ASD (i.e., *quality* time). Parent-child quality time has been characterized in the literature broadly as time during which caregivers and children feel closeness, connection, and togetherness (Fallon & Bowles, 1997). This lack of research exploring positive aspects of the parent-child relationship has been posited by scholars as due to a history of inaccurate and stigmatizing theories implying parents’ lack of warmth is responsible for ASD in their children (for a discussion, see Crowell et al., 2019).

Thus, research on quality time has been restricted to families of neurotypical youth. Outcomes point to quality time during shared activities as being a key factor in positive caregiver involvement in the parent-child relationship (Hipwell et al., 2008). Indeed, studies of neurotypical families have shown that positive caregiver behavior during shared interactions (e.g., caregiver involvement, responsiveness, warmth) is associated with higher rates of child prosocial behavior (Batool & Lewis, 2020; Kawabata et al., 2011). Further, researchers examining family leisure activities (i.e., time during which family members spend time together and communicate; Zabriskie & McCormick, 2001) have found support for both involvement and satisfaction in family leisure time playing significant roles in family functioning (Smith et al., 2009; Townsend & Zabriskie, 2010). Theoretical models (e.g., Hastings et al., 2002) have proposed that parenting stress negatively impacts caregiver behavior and caregiving ability. Alternatively, a reimagining of the family stress model (Conger et al., 1994; 2002) by Schiltz and colleagues (2018) recognizes that the familial challenges of raising a child with ASD may have the same conceptual pathway in influencing caregiver functioning (e.g., stress) and behavior (e.g., caregiver involvement) as family financial strain. Indeed, in a study of 150 families of children with ASD, Hickey and colleagues (2020) found evidence that maternal parenting stress is strongly linked to decreased warmth and increased criticism in parent-child interactions. Therefore, there is a likely connection among raising a child with ASD, caregiver stress, and the presence of parent-child quality time. An examination of additional important factors that have been identified in the child and family literature (e.g., shared enjoyment and synchronicity during parent-child quality time) in the context of parenting stress would extend this current body of work.

Early research of parent-child relationships in youth with ASD explored attachment behavior and suggested caregiver sensitivity and parent-child synchronicity lay the foundation for the development of secure attachment and later child communication skills (Siller & Sigman, 2002). More recent research has found that shared activities and caregiver involvement are also important factors in the development of strong parent-child relationships (Keller et al., 2014; Walton & Tiede, 2020). However, caregivers of youth with ASD may encounter increased barriers to participating in shared activities with their child. Research has demonstrated that children with ASD may participate in leisure activities, such as mealtimes and social play, less frequently than their neurotypical peers (Hochhauser & Yeger, 2010). While researchers have identified that children with ASD often engage in activities alone (e.g., reading, using the computer, playing video games; Reynolds et al., 2011), the assumption that simply increasing child and caregiver quality time will promote positive family outcomes may not be the case. In fact, Walton (2019) reported that decreased caregiver *satisfaction* with family leisure activities, rather than decreased *involvement* in family leisure activities, was related to poorer family functioning among families of youth with ASD. As such, further exploration of shared activities and other factors relating to quality time (e.g., caregiver *enjoyment* of shared activities) is needed to promote positive outcomes among ASD families.

While this growing body of work highlights the importance of quality family time for positive outcomes among families of youth with ASD, little is known about the specific child and caregiver characteristics that may relate to parent-child quality time. As social expectations and influences evolve across child development, child age has been implicated in research relating to quality time and shared activities among youth with ASD. In a comparison of three age cohorts (ages 5–6; ages 7–9; and ages 10–12), Little and colleagues (2015) found that younger children tended to participate more frequently in family activities in the home compared to older children, who engaged more frequently in activities in the community. While this trajectory appears to align with that of typically developing children, parent-child quality time among families of youth with ASD may be distinct given increased caregiver involvement in education (Yan et al., 2022) and intervention (Musetti et al., 2021) processes throughout childhood and adolescence. Further, the distinction between solitary home activities and shared activities remains critical; in an in-depth exploration of activity participation across two youth cohorts (age 5; ages 9–10), Simpson and colleagues (2018) found that caregivers of older children with ASD desired decreased involvement in screen-based activities and increased involvement in shared family activities. Neither of these studies, nor any known to date, have considered the role of caregiver wellbeing across child age cohorts in the experience of family quality time. Indeed, ASD research has suggested child age may influence caregiver stress, though findings have been inconsistent. While some studies have reported higher stress in caregivers of younger children (e.g., Barker et al., 2011), others have suggested caregiver stress spikes in middle childhood (Orr et al., 1993). Moreover, an examination of four age cohorts of children with ASD revealed no significant differences in parenting stress across developmental groups (McStay et al., 2014). Still, assessing age-related differences in both caregiver stress and quality time may help clarify these constructs across important developmental transitions.

The current study seeks to examine the association between parenting stress and factors implicated in parent-child quality time among caregivers of children with ASD. Data was collected from a nation-wide sample of youth with ASD. It is important to note that this data was collected during the COVID-19 pandemic prior to the availability of vaccines for most adults and children (December 2020). Large-scale shutdowns resulted in caregivers managing the bulk of their child’s schooling, meals, and other activities throughout the week (Colizzi et al., 2020; Alhuzimi, 2021). It was predicted that elevated caregiver stress would be associated with less time spent in parent-child shared activities (on the weekday and weekend), and decreased enjoyment in shared activities. Additionally, it was predicted that caregivers experiencing clinically significant levels of parenting stress would be less likely to report the presence of parent-child shared activities and the presence of synchronicity with their child compared to caregivers below the clinical threshold. Finally, we explored the caregiver stress, the presence of shared activities, and the presence of synchronicity across three developmentally distinct age cohorts. It was predicted that more caregivers of younger children would report the presence of shared activities and synchronicity than caregivers of older children.

## Method

The present study recruited participants using the Simons Foundation Powering Autism Research for Knowledge (SPARK) Research Match service. SPARK recruits families of children with ASD diagnosed at certified autism research centers in the U.S. Consenting families are contacted when new studies are approved through the SPARK scientific committee. Families volunteer to complete assessment measures online, their responses are deidentified, and the deidentified data are provided to the collaborating researchers. The ongoing SPARK recruitment has engaged a network of 50,000 individuals with ASD and their family members. Data collection for the current project took place in December 2020.

### Participants

Eligibility criteria for caregivers included (1) must be the legal guardian of the enrolled youth, (2) at least 18 years of age, (3) who can read and write in English, and (4) had previously completed measures through SPARK on their children’s social communication and restricted/repetitive behaviors. Eligibility criteria for youth included (1) must have a diagnosis of ASD, (2) be between the ages of 0 to 17 years, and (3) who was previously registered through SPARK. A total of 582 caregivers of youth with ASD (ages 4–17 years; *M* = 10.81, *SD* = 4.50) consented to the study and initiated the online assessment. Of those consenting adults, only 511 completed the assessment battery and were included in the current study analyses (see Table 1 for full demographic information). The majority of caregivers were married (*N* = 353; 69.2%) and were between the ages of 22–66 years (*M* = 40.4, *SD* = 7.73). Participating caregivers consisted of mostly mothers (*N* = 463; 91.1%). The majority of children were male (*N* = 393; 77.2%) and White (77.0%). Participating caregivers lived in the United States and spoke English. Participants received compensation for their time.

**Table 1:** *Demographic Characteristics of Sample*

		*M*	*SD*		*n*	Percentile
Child Gender						
	Male Female Transgender Nonbinary Total			393 106 6 4 509		77.2 20.8 1.2 0.7
Child Age (years)		10.8	4.5			
Child Ethnicity						
	Hispanic/Latinx Asian Black/African American American Indian/Alaska Native Native Hawaiian/Other Pacific Islander White Other			90 24 50 18 6 448 22		15.5 4.1 8.6 3.1 1.0 77.0 3.8
Number of Family Members		4	1.4			
Caregiver Current Age		40.4	7.7			
Caregiver Relationship to Child						
	Mother Father Grandmother Aunt Stepmother Foster Caregiver/Guardian Other Total			463 31 6 1 2 3 2 508		91.1 6.1 1.2 0.2 0.4 0.6 0.4
Caregiver Marital Status						
	Married/Domestic Partnership Divorced Widowed Single/Never Married Total			353 95 4 58 510		69.2 18.6 0.8 11.4
Current Yearly Household Income						
	Less than $20,999 $21,000- $35,999 $36,000- $50,999 $51,000- $65,999 $66,000- $80,999 $81,000- $100,999 $101,000- $130,999 $131,000- $160,999 Over $161,000 Total			81 78 74 50 56 58 38 30 41 506		16.0 15.4 14.6 9.9 11.1 11.5 7.5 5.9 8.1

### Measures

#### Parenting Stress

Parenting stress was measured using the Parenting Stress Index-Short Form (PSI-SF), which is a 36-item survey (Abidin, 1995). Questions such as, “I often have the feeling that I cannot handle things very well,” and “I feel trapped by my responsibilities as a parent,” are scored on a Likert-scale from 1 - *strongly disagree*, to 5 - *strongly agree*. Given that the original PSI-SF factor structure has not performed well in samples of autistic children, the current researchers employed a revised structure validated among caregivers of youth with ASD (Cronbach’s α of 0.79 − 0.86; Zaidman-Zait et al., 2011). Responses on the measure were summed to generate a total score and the revised PSI-SF factors: General Distress, Parenting Distress, Reward Parent, Child Demandingness, and Difficult Child. Internal consistency reliability was high for the total score and all subscales (General Distress *α* = 0.82; Parenting Distress *α* = 0.85; Reward Parent *α* = 0.84; Child Demandingness *α* = 0.75; Difficult Child *α* = 0.78). We also created a dichotomous measure of ‘clinically significant’ parenting stress using the recommended cut-off score for the PSI-SF (Abidin, 1995). The cut-off score distinguishes caregivers meeting clinically significant levels of parenting stress and has been used in samples of caregivers of youth with ASD (Zaidman-Zait et al., 2010). The cut-off has demonstrated utility in identifying high-risk families who may need additional services and thus has particular applicability in clinical settings (e.g., Barroso et al., 2016). Examining the relationship between the derived clinical cut-off and novel aspects of family quality time may provide direction for clinicians monitoring and addressing parenting stress among families of youth with ASD.

### Quality Time

Quality time was assessed using single items from a survey developed by the current research team. The current project utilized six items: time in shared activities (weekend day, weekday), caregiver and child enjoyment of shared activities, the presence of shared activities, and the presence of synchronicity. Caregivers were asked to report how many hours they spend engaging in the same activity with their child on a typical weekday and weekend day on a scale of 0–25 h. Caregivers were also asked to rate *how often* they are enjoying themselves and how often they perceive their child to be enjoying themselves during shared activities using a percentage value (from 0 to 100% of the time).

Presence of shared activities between the child and caregiver was operationalized by asking the caregiver to respond (*yes*/*no*) to the question, “Do you and your child/dependent spend time together doing shared activities?” If the participant responded yes, they were then prompted to rank from a selection of 15 common activities in which they spend time with their child (1 = *most time spent* to 15 = *least time spent*). Synchronicity between caregiver and child has been defined as patterns of reciprocation or ‘give-and-take’ behaviors (Barber et al., 2001). The current study operationalized synchronicity by asking the caregiver to respond (*yes*/*no*) to the question, “Do you ever feel like you are in-sync with your child/dependent (thinking about the same things, understanding his/her wants/needs, ‘in tune’ with one another)?”

### Analysis Plan

We examined significant caregiver stress using the recommended clinical cut-off variable derived from the PSI-SF total raw score (Abidin, 1995). Comparisons between demographic characteristics were performed using independent samples *t*-tests and chi-square analyses to determine if differences arose between families with or without clinically significant stress. Pearson correlations were used to assess the relationship between parenting stress, time caregivers spent engaging in the same activity as their child on a typical weekday and weekend day (*time in shared activities*), and caregiver reports of their and their child’s enjoyment of shared activities. Chi-square tests of independence were used to compare caregivers with clinically significant stress and caregivers without clinically significant stress on (a) the presence of shared activities with their child, and (b) the presence of synchronicity (*feeling in-sync*) with their child. Clinically significant parenting stress, presence of synchronicity, and presence of shared activities were compared across child age groups. We generated age groups based off Center for Disease Control and Prevention guidelines to capture early childhood (< age 6), middle childhood/early puberty (ages 6–12), and adolescence (ages 13–17). Finally, explorations of activities caregivers engaged in with their children were calculated via a frequency count. There were few missing data on PSI-SF individual items, with no more than 3 missing respondents on any single item (0.6% or less missing). Missing values for individual items (n = 33) were imputed as the respondent’s mean for the item’s subscale. Caregivers with missing data on the quality time items (presence of shared activities, presence of synchronicity, time in shared activities, and enjoyment of shared activities) were excluded pairwise.

## Results

### Demographics

Caregivers above and below the clinical threshold on the PSI-SF were compared on demographic variables (i.e., child age, gender, ethnicity/race, household income, relationship to child in treatment). The only significant difference that arose between demographic characteristics was child race, *χ*^2^(1) = 13.0, *p* < .001. This finding indicates that there were proportionally more caregivers of White children reporting clinically significant stress than caregivers of non-White children. All other demographic variables were not significantly different between groups (see Table 1 for all demographic variables).

### Parenting Stress, Parent-Child Time in Shared Activities, and Enjoyment of Shared Activities

Pearson correlations were run to assess the relationship between parenting stress and quality time spent with children with ASD (see Table 2). The number of hours spent in which the caregiver and child were engaging in the same activity on a typical weekday was significantly negatively correlated with two of the five PSI-SF subscales: Parenting Distress (*r* = − .10, *p* < .05) and Child Demandingness (*r* = − .11, *p* < .05). There were no significant associations found among between the weekday time engaging in shared activities and the General Distress, Rewards Parent, or Difficult Child subscales. Therefore, caregivers who endorsed more distress unique to the parenting role and held more perceptions of their caregiving role as difficult reported less time spent engaging in shared activities with their child on the weekday.

**Table 2:** *Descriptive Statistics and Correlations for Parenting Stress and Quality Time Variables*

Variable	*n*	*M*	SD		1	2	3		4		5		6		7			8		9
1. PSI Total	511	97.45	23.69																	
2. PSI GD	511	21.54	6.56	0.68**																
3. PSI PD	511	13.88	5.23	0.78**		0.58**														
4. PSI RP	511	18.94	6.70	0.74**		0.48**		0.45**												
5. PSI CD	511	14.90	4.45	0.79**		0.44**		0.62**		0.62**										
6. PSI DC	511	20.82	5.67	0.62**		0.41**		0.39**		0.68**		0.65**								
7. Time (Weekend)	509	6.52	5.84	− 0.07		0.02		− 0.07		− 0.09		− 0.07		0.01						
8. Time (Weekday)	510	5.41	5.71	− 0.10*		0.02		− 0.10*		− 0.08*		− 0.11*		− 0.02		0.82**				
9. Child Enjoyment	483	71.78	23.16	− 0.33**		− 0.14*		− 0.19**		− 0.39**		− 0.25**		− 0.22**		0.20**	0.09			
10. Caregiver Enjoyment	478	73.64	22.32	− 0.41**		− 0.20**		− 0.29**		− 0.39**		− 0.36**		− 0.23**		0.15**	0.08		0.60**	

*Note*: PSI scores were derived from the Parenting Stress Index-Short Form. Subscale abbreviations are as follows: PSI GD = General Distress subscale; PSI PD = Parenting Distress subscale; PSI RP = Rewards Parent subscale; PSI CD = Child Demandingness subscale; PSI DC = Difficult Child subscale (Zaidman-Zait et al., 2011). Time indicates parent estimated hours spent engaging in the same activity with their child on a typical weekday or weekend day. Parent and Child Enjoyment were derived from parent estimates of the amount of time parents and children are enjoying themselves during shared activities (rated from 0–100% of the time)

**. Correlation is significant is significant at the 0.001 level

*. Correlation is significant at the 0.05 level

In contrast to weekday results, time caregivers spent engaging in shared activities with their child on a typical weekend day was only significantly associated with the Reward Parent subscale on the PSI-SF (*r* = − .09, *p* < .05). Caregivers who reported their interactions with their child to be less rewarding and endorsed fewer elements of positive parent-child interactions (i.e., higher scores on the Reward Parent subscale) tended to spend less time engaging in shared activities with their child on the weekend. To further examine these constructs, we used an independent *t-*test to compare parent-reported time spent in shared activities on a typical weekday vs. weekend. Caregivers, on average, reported spending significantly more time in shared activities on a typical weekend day than on a weekday, *t*(1017) = -3.06, *p* < .05 (see Table 2).

Caregivers reported “how often” they enjoy themselves and their perception of how often their child to enjoys themself during parent-child shared activities (0–100% of the time). Child enjoyment was significantly negatively correlated with all PSI subscales. Similarly, caregiver enjoyment was significantly negatively correlated with all PSI subscales (see Table 2). Caregiver enjoyment during shared activities was strongly associated with caregiver perception of child enjoyment (*r* = .60, *p* < .001; see Table 2). Parent-child time spent in shared activities on a typical weekend day was significantly, positively associated with both child enjoyment (*r* = .20, *p* < .001) and caregiver enjoyment (*r* = .15, *p* < .001). Child enjoyment (M = 71.78, SD = 22.16) and caregiver enjoyment (M = 73.64, SD = 22.32) during shared interactions did not differ significantly, *t*(959) = -1.27, *p* = .21. Overall, higher rates of general distress and distress specific to the parenting role, interactions with their child, and child characteristics were associated with less caregiver enjoyment and perceived child enjoyment during shared activities.

### Parenting Stress, Shared Activities, and Synchronicity

**Fig. 1:**
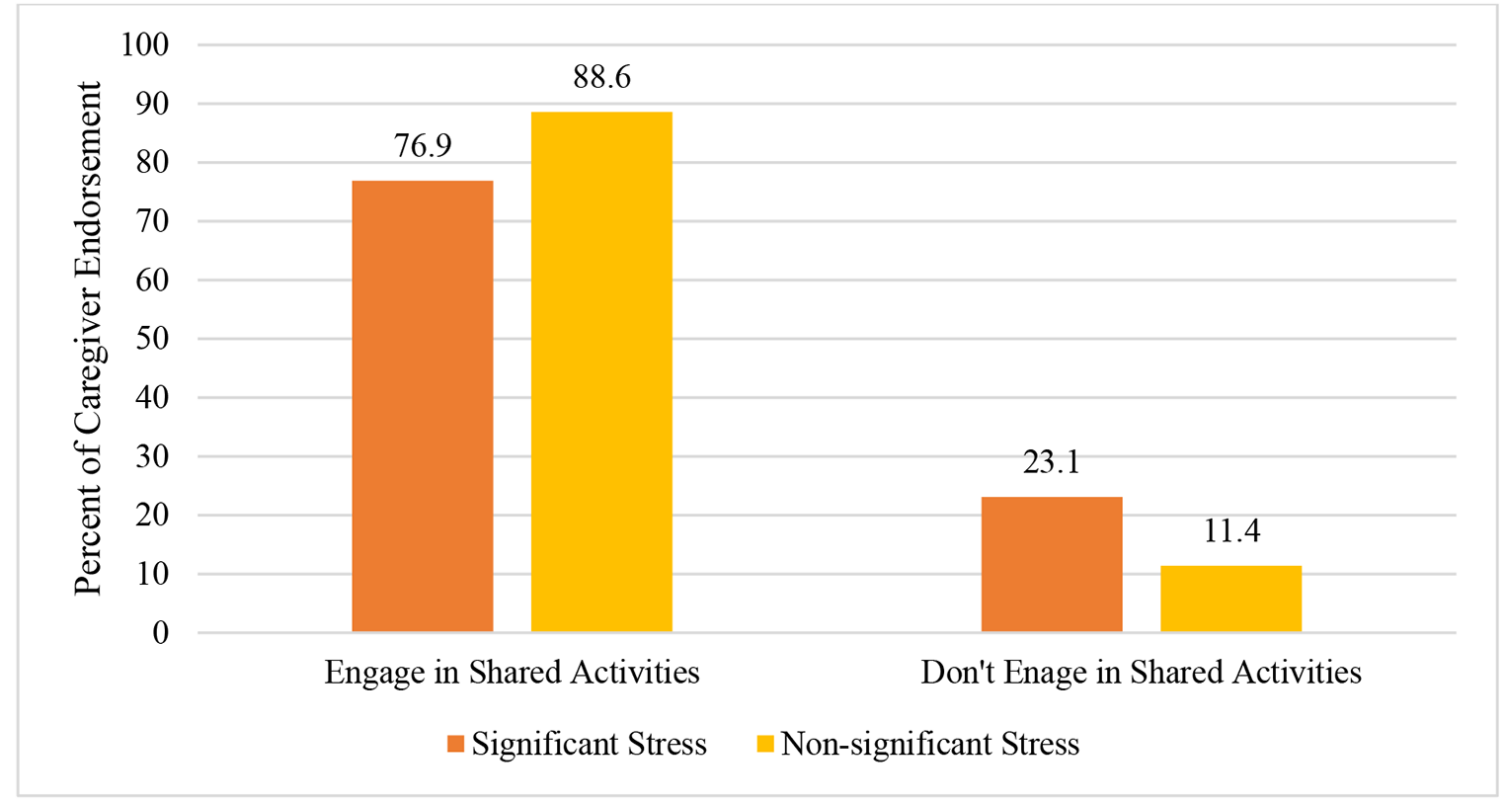
*Parenting Stress and Shared Activities***(***Note.* Significant stress was determined by the clinical cut-off score on the PSI-SF. Chi square test of parenting stress and shared activities; *X*^2^ (1) = 12.37, *p* < .001)

Chi-square tests were performed to assess the relation between significant stress as determined by PSI-SF ratings and the presence of shared activities and synchronicity. The relation between parenting stress and the presence shared activities was significant, *X*^2^ (1) = 12.37, *p* < .001 (see Fig. 1). About one-fourth of caregivers experiencing clinically significant stress (23.1%) reported not engaging in any shared activities with their child, whereas only 11.4% of caregivers without clinically significant stress reported not engaging in shared activities. Thus, 88.6% of caregivers below the clinical threshold reported engaging in shared activities while only 76.9% of caregivers above the clinical threshold endorsed shared activities. Results indicated there was a greater proportion of caregivers with lower stress that engaged in shared activities with their child compared to caregivers with clinical levels of stress.

**Fig. 2:**
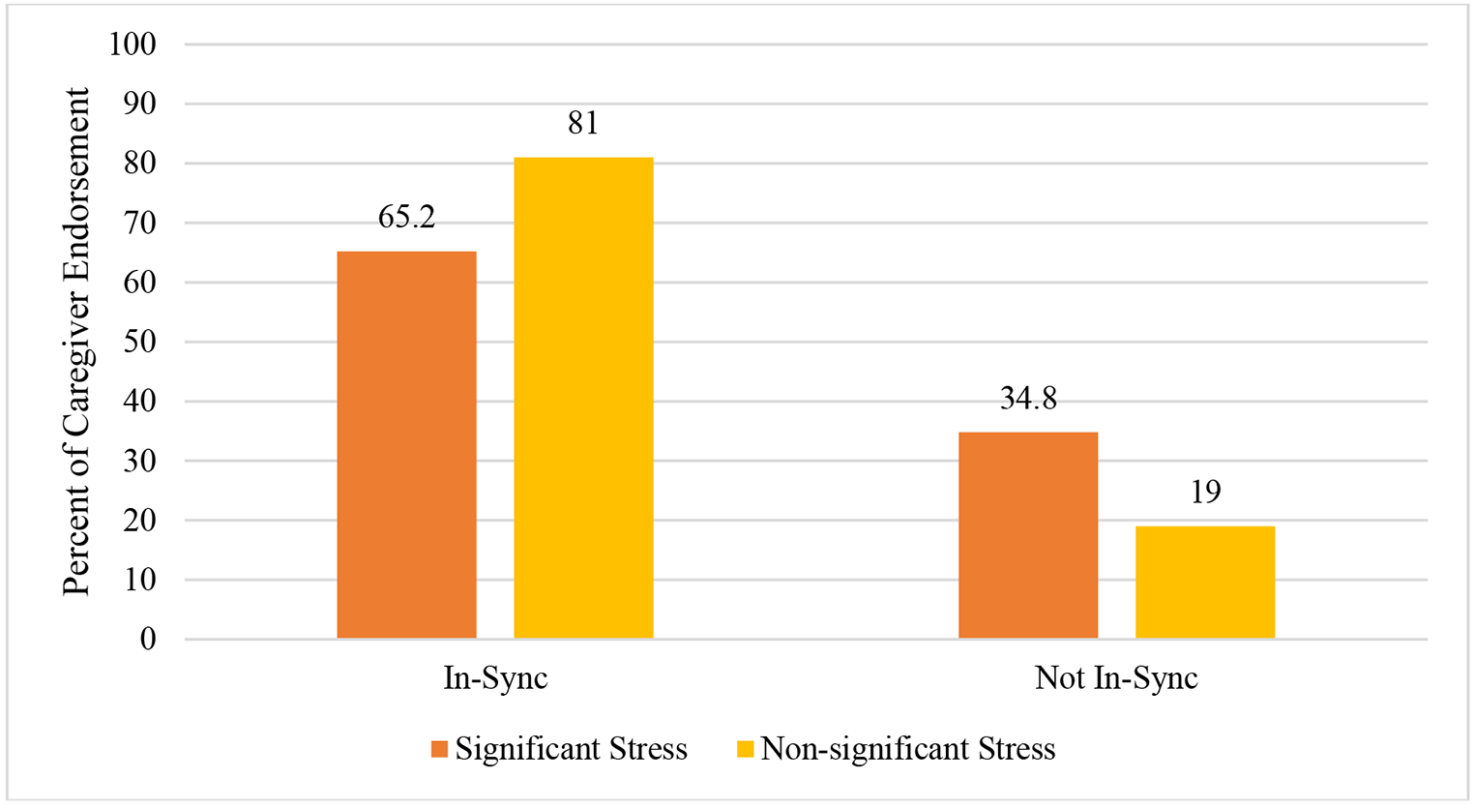
*Parenting Stress and Synchronicity***(***Note.* Significant stress was determined by the clinical cut-off score on the PSI-SF. Chi square test of parenting stress and synchronicity with child; *X*^*2*^ (1) = 16.32,*p* < .001.)

The relation between parenting stress and the presence of synchronicity was also significant, *X*^2^ (1) = 16.32, *p* < .001 (see Fig. 2). Approximately one-third of caregivers experiencing clinically significant stress (34.8%) reported not being in-sync with their child, whereas 19.0% of caregivers without clinically significant stress reported not being in-sync. Stated another way, 65.2% of caregivers above the clinical threshold reported synchronicity with their child, while 81.0% of those below the clinical threshold endorsed synchronicity. Results demonstrate a greater proportion of caregivers with lower stress endorse being in-sync with their child compared to caregivers with clinically high stress.

Parenting stress, shared activities, and synchronicity were compared across age groups using chi-square analyses. Results indicate that proportionally more caregivers of young children (younger than 6 years) endorse the presence shared activities with their child compared to caregivers of older children (ages 13–17 years), *X*^2^ (1) = 16.26, *p* < .001 (see Table 3). No significant differences emerged for parenting stress or the presence of synchronicity across age groups.

**Table 3:** *Comparisons of Stress, Synchronicity, and Shared Activities Across Child Age Groups*

	Total		Ages < 6		Ages 6–12		Ages 13–17				
	* N*	%	*N*	%	*N*	%	*N*	%		*χ* ^2^	*p*
Stress Non-Clinical Stress Clinically Significant Stress	509 288 221	56.6 43.4	158 93 65	58.9 41.1	187 107 80	57.2 42.6	164 88 76	53.7 46.3		0.94	0.63
Synchronicity Yes No	508 377 131	74.2 25.8	157 122 35	77.7 22.3	187 139 48	74.3 25.7	164 116 48	70.7 29.3		2.04	0.36
Shared Activities Yes No	508 424 84	83.5 16.5	158 145 13	91.8 8.2	186 155 31	83.3 16.7	164 124 40	75.6 24.4		15.24	< 0.001

*Note.* Clinically significant stress was determined by the clinical cut-off score on the PSI-SF.

### Shared Activities Between Caregivers and Youth with ASD

When asked to rank which activities caregivers spend the most time doing with their children, the three most commonly top ranked activities were eating, schoolwork, and talking. Seventy-two reporting caregivers (18%) ranked eating as their most frequent shared activity, followed by 62 caregivers (17%) selecting schoolwork, and 61 caregivers (16%) selecting talking.

## Discussion

The primary aim of the current study was to explore the relationship between parenting stress, quality time, and shared activities among caregivers of youth with ASD. As predicted, results indicated several indices of parenting stress were negatively associated with the amount of time caregivers spent with their child in shared parent-child activities during a typical weekday and weekend day. Interestingly, this relationship differed by PSI-SF subscale. While higher rates of both Parenting Distress and Child Demandingness were associated with less time spent in shared activities on a typical weekday, only Reward Parent was associated with less time spent in shared activities on a typical weekend day. As such, it appears higher rates of distress with the parenting role and perceptions of one’s child as more difficult than expected are associated with less time in shared activities throughout the week, while reduced endorsement of positive aspects of the parent-child relationship are associated with less time on weekends. Though literature on shared activities for ASD families throughout the week is limited, Walton (2019) employed the core and balance model (Zabriskie & McCormick, 2001) in a study of family leisure activity among caregivers of youth with ASD. “Core” activities refer to routine activities for the family (e.g., playing games inside), while “balance” activities may involve non-routine, special events. It stands to reason these categories may map well onto weekday and weekend shared activities, as “core” activities likely occur throughout the week and “balance” activities may cluster towards weekends when children are not in school. While this study concluded families of youth with ASD are involved in core and balance activities at similar rates as families of neurotypical youth, the researchers did not assess parenting stress. Our findings suggest increased rates of parenting stress may relate to less involvement in parent-child shared activities.

Further, parenting stress was negatively associated with both caregiver and child enjoyment during shared activities. Together, these findings suggest that caregivers with higher stress spend less time engaging in shared activities with their child during the week, and they perceive their interactions to be less enjoyable for both themself and their child. Results also revealed that caregivers with clinically significant levels of stress reported engaging in shared activities and feeling in-sync with their child less often than caregivers without clinically significant stress. These results align with recent research by Hickey and colleagues (2020) demonstrating the association between greater parenting stress and negative parent-child relationship quality. The current exploration extends these findings by implicating factors associated with quality time (i.e., caregiver perceptions of shared enjoyment and synchronicity) in this relationship. Further, by examining parenting stress at the factor-level, we found that distress associated with parenting, difficulties with child behavior, and reduced positive perceptions of the parent-child relationship are uniquely associated with less time spent engaging in shared activities. Despite limited extant research, it is likely that quality time plays an important role in parent-child relationships among families of youth with ASD. Further exploration of quality time in the context of parenting stress may provide researchers opportunities to foster positive family outcomes across child development.

We compared parenting stress, quality time, and synchronicity across child age groups (i.e., < 6 years; 6–12 years; 13–17 years). No differences were found across ages for measures of parenting stress and quality time; but significant differences arose for quality time across the age groups. These results suggest that caregivers of younger children (< 6 years) spend quality time with their children more frequently than caregivers of older children (13–17 years). Although the literature on parent-child quality time across development in youth with ASD is sparse, these results align closely with past research with neurotypical youth. Previous studies have identified a pattern of increased autonomy and interactions with peers and decreased interactions with family over the adolescent years (Larson et al., 1996; Larson & Richards, 1991). For adolescents with ASD, the importance of developing self-determination and autonomy increases as children approach transitional years (White et al., 2021). Nonetheless, family support, acceptance, and involvement remain important during transitional periods of adolescence and early adulthood, as these qualities are broadly associated with higher self-esteem, positive adult relationships, and greater educational involvement (Smith & Anderson, 2014). Proportionally fewer caregivers endorsing the presence of shared activities with their adolescents, although normative compared to neurotypical youth, may also reflect the limited resources available to guide caregivers with transition-aged youth on the spectrum (Cheak-Zamora et al., 2020).

We also explored types of shared activities among caregivers and youth with ASD. Caregivers reported the activities they spent the most time doing with their child were eating, schoolwork, and talking. Examining shared activities in this population is important because, although children with ASD often require more supervision and support than neurotypical children, this time spent together may not elicit shared enjoyment in caregivers and children. Mealtimes, for example, may be a source of family stress if a child experiences eating/feeding challenges (Ismail et al., 2020). Given that this data was collected during the COVID-19 pandemic, it is possible that children were spending more time at home and required more support from their caregivers to complete schoolwork. This could lead to more caregiving stress considering many educational and therapeutic services typically accessed by youth with ASD may not have been available due to COVID-19 shut-downs (Alhuzimi, 2021). Indeed, caregiver and child satisfaction with shared activities, rather than rate of involvement, have been linked to positive family functioning (Walton, 2019). Quality time spent between caregivers and children may include talking about emotions and problem-solving. Caregivers report that talking through difficult situations with their child with ASD helps them regulate down when overstimulated or overwhelmed (Glazzard & Overall, 2012). Future research is needed to explore the impact of shared activities in parent-child interactions of youth with ASD and how these may strengthen the parent-child relationship.

We also explored demographic differences (e.g., race/ethnicity, income, child age) among families above and below the clinical threshold for caregiving stress on the PSI-SF. Findings revealed significantly more caregivers of White children endorsing clinical levels of stress compared to caregivers of non-White youth. While there is a substantial body of work documenting higher rates of caregiving stress among caregivers of youth with ASD, the research on this relationship across racial groups is mixed. Past research has documented greater levels of caregiving stress among Black caregivers of youth with ASD compared to White caregivers (Williams et al., 2019), and less caregiving stress among Latinx families compared to other groups (Valicenti-McDermott et al., 2015). Further, emerging research on racial/ethnic differences in family resilience has found a unique relationship between these variables in Black families; specifically, more resilience is strongly associated with less caregiving stress (Kim et al., 2020). Notably, past literature has also highlighted service disparities among non-White families of youth with ASD, and these pertain to both child therapeutic services and caregiver self-care resources (Mandell et al., 2002; Zuckerman et al., 2014). Likewise, barriers in accessing ASD services, such as stigma and lack of comprehensive information, has been implicated as a source of stress for caregivers of color (Stahmer et al., 2019). Although not explicitly explored in the current analyses, it is possible that our participating families’ engagement with the SPARK project speaks to their connection to local services. Thus, both family and community influences may be protective against stress for families of color in our current sample.

While previous research has established that caregivers of children with ASD experience higher rates of clinically significant stress than caregivers of neurotypical children, understanding how this stress affects the parent-child relationship has yet to be determined (Ingersoll & Hambrick, 2011). Our findings suggest parenting stress associated specifically parent-child dynamics (i.e., distress relating to parenting demands, perceptions of their child as difficult, reduced endorsement of positive parent-child characteristics) may interfere with parent-child quality time. Indeed, past studies have identified that caregivers of youth with ASD may also experience caregiver burden, which is associated with depleted caregiving abilities (Burke & Heller, 2016). The current findings contribute to this understanding by highlighting the differences in quality time spent between caregivers of children with ASD with and without clinically significant stress. Considering ASD is characterized by difficulties in social communication and interaction, youth often need more hands-on support, which in turn places more demands on caregivers (Burke & Heller, 2016). These demands and higher stress may leave caregivers with a lower capacity for initiating or maintaining positive interactions with their child during quality time (Osborne & Reed, 2008). In turn, this may contribute to global family stress, as decreased relationship quality may negatively impact both caregiver and child wellbeing in families of youth with ASD (Keller et al., 2014; Siller et al., 2013.).

The present study, although novel, had several limitations. First, quality time data was derived from single item responses on a questionnaire developed by the research team. Thus, reliability metrics for quality time responses have yet to be established. Additionally, the use of dichotomized metrics (i.e., presence of synchronicity, presence of shared activities, clinically significant stress) provide descriptive, rather than predictive, information about the relationship among these variables. The observed correlations do not prove causation or directionality among parenting stress and quality time factors. Nonetheless, the current use of single item metrics to assess quality time yield exploratory information about the landscape of quality time among families with ASD. Further, health service research has demonstrated that single-item ratings can perform well as a reliable measure (Macias et al., 2015). In addition, data was only collected from caregivers of youth with ASD, rather than children themselves. It is possible that caregivers inaccurately assessed their child’s level of enjoyment during shared interactions and hearing directly from young individuals with ASD may yield more valuable information about this burgeoning topic. In step with recent efforts encouraging researchers to center the voices of individuals with ASD (Fletcher-Watson et al., 2019), future work should utilize self-report from youth with ASD. Further, utilizing a comparison sample of neurotypical families may help parse apart factors unique to parent-child relationships among families of youth with ASD.

Considering these limitations, the current findings still underscore the importance of quality time, shared enjoyment, and synchronicity among caregivers and children with ASD. Increased parenting stress may prevent caregivers from spending quality time with their children (Burke & Heller, 2016; Osborne & Reed, 2008); alternatively, reduced time in shared activities and enjoyment of shared activities may contribute to increased rates of parenting stress. Moreover, the development of parenting stress and the reduction of parent-child quality time may be cyclical in nature (e.g., increased stress leads to lack of quality time spent together which leads to increased stress). It is most probable that the link between parenting stress and the parent-child relationship is complex and involves additional variables that were not explored in the current study. It would be beneficial for future research efforts to examine additional factors that influence this relationship such as other family-related variables (e.g., marital satisfaction, caregiver psychopathology), child perceptions and experiences, access to resources and services, and child-related factors (e.g., ASD symptom severity, disruptive behavior; Zaidman-Zait et al., 2014). These findings also support future intervention research. Considering the results of the PSI-SF subscale analyses, parent training efforts targeting the parent-child relationship may be particularly instrumental in reducing parenting stress and improving family interactions. Early interventions emphasizing responsive parenting have demonstrated the ability to improve responsive communication, joint engagement, and caregiver psychological wellbeing (e.g., McConachie & Diggle, 2006; Siller et al., 2013). Improvements in the caregiving environment, specifically parenting behavior and child attachment, have also been shown to improve child symptoms (e.g., executive functioning) associated with parenting stress (Bernier et al., 2012; Tsermentseli & Kouklari, 2021). It is recommended that researchers continue to explore the efficacy of evidence-based interventions focused on improving the parent-child relationship (e.g., Parent-Child Interaction Therapy) and reducing parenting stress (e.g., mindfulness-based skills programs) to assess quality time and global family functioning outcomes over time.

In sum, a significant relation exists between parenting stress and quality time spent among caregivers and their children with ASD. Caregivers under higher levels of stress tend to spend less time with their children, are less likely to endorse synchronicity with their child, and their interactions tend to be less enjoyable for both parties. It is important to further examine how stress and other familial factors impact parent-child interactions, as quality time is implicated in child development and positive outcomes for families of youth with ASD.
